# Static Scanning Tunneling Microscopy Images Reveal the Mechanism of Supramolecular Polymerization of an Oligopyridine on Graphite

**DOI:** 10.1002/anie.202117580

**Published:** 2022-02-21

**Authors:** Felix D. Goll, Gerhard Taubmann, Ulrich Ziener

**Affiliations:** ^1^ Ulm University Institute of Organic Chemistry III Albert-Einstein-Allee 11 89081 Ulm Germany; ^2^ Ulm University Institute of Theoretical Chemistry Albert-Einstein-Allee 11 89081 Ulm Germany

**Keywords:** Chain Growth Polymerization, Oligopyridine, Reaction Mechanisms, Scanning Tunneling Microscopy, Self-Assembly

## Abstract

Supramolecular polymerization of a donor–acceptor bisterpyridine (BTP) equipped with an electron‐rich carbazole unit is observed by scanning tunneling microscopy (STM) at the highly oriented pyrolytic graphite (HOPG)|solution interface. It is shown that two‐dimensional crystals of supramolecular (co)polymers are formed by chain growth polymerization, which in turn can be described by copolymerization statistics. From concentration‐dependent measurements, derived copolymerization parameters and DFT calculations, a mechanism for self‐assembly is developed that suggests a kinetically driven polymerization process in combination with thermodynamically controlled crystallization.

## Graphical abstract

To be or not to be: The pressing question of the fundamental mechanism of two‐dimensional self‐assembly processes, namely that of thermodynamic or kinetic control, is demonstrated using the example of a donor–acceptor oligopyridine on graphite. A statistical analysis of STM images and DFT calculations suggest that there is a significant contribution of kinetics similar to copolymerization in bulk.

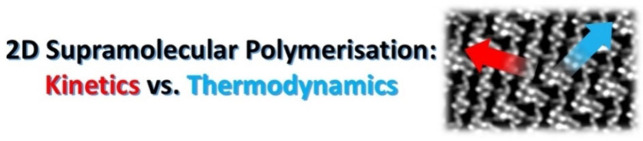

Supramolecular polymerization by self‐assembly has proven to be a powerful method to create functional materials with unparalleled mechanical properties and potential applications in, for example, electronics and biomedicine.^[1]^ In order to control the process of polymer formation, a good understanding of the mechanism behind the self‐processes is essential. There are many reports devoted to elucidating these mechanisms. They are often based on thermodynamic models and fewer reports are known of kinetically controlled supramolecular polymers.^[2]^ This becomes even clearer in surface‐confined supramolecular polymerization because of the strong substrate‐adsorbate interactions and adsorbate‐adsorbate attractive forces in two dimensions (2D). Typically, the formation of 2D assemblies is described as crystallization under thermodynamic control.^[3]^ But there are also examples of kinetic control, e.g., the self‐assembly of 10,12‐pentacosadiynoic acid (PCDA) under confinement on a “nanoshaved” structured surface^[4]^ or the phase transition between pseudopolymorphs of octadecylcarbamic acid tetradecyl ester in analogy to Ostwald's rule of stages.^[5]^

Scanning probe techniques such as scanning tunneling microscopy (STM) are standard methods for the characterization of 2D structures. There are several examples of supramolecular polymers visualized by STM.^[6]^ In addition, many small molecules self‐assemble to form supramolecular 2D polymers, driven by crystallization. Bisterpyridines (BTPs) are a typical class of compounds that readily self‐assemble on various substrates, which can be explained on a thermodynamic basis.^[7]^ However, there are known kinetic models for one‐dimensional (1D) polymerization in bulk, which in the case of copolymers lead to defined distributions of polymerization degrees or sequence lengths that also apply to supramolecular polymers in bulk. The question arises whether the 2D self‐assembly can be described by a quantitative statistical model of 1D polymerization despite the confined space and the strong ordering forces.

Here we report on the supramolecular (pseudo‐co‐)polymerization of a donor‐substituted BTP (2,2′‐BTPCz) (Figure 1) on the surface, which leads to a distribution of different sequence lengths even within domains. A statistical analysis of the static STM images shows that this distribution perfectly follows the kinetic penultimate model of 1D chain growth copolymerization in bulk. The arrangement orthogonal to the direction of polymerization can be explained by crystallization under thermodynamic control. The concentration has a direct influence on the sequence length distribution due to subtle adsorbate‐adsorbate interactions.

**Figure 1:**
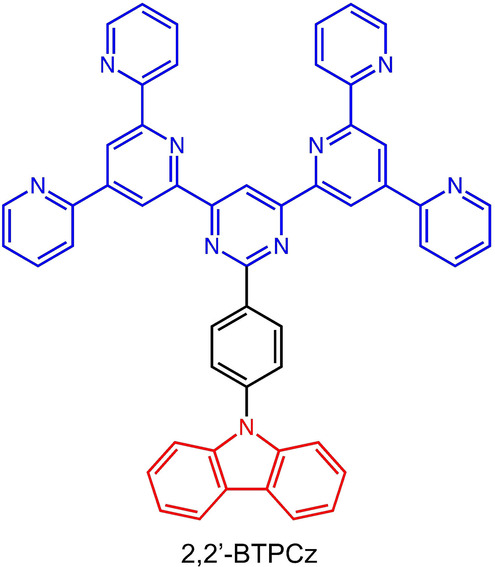
Donor–acceptor molecule 2,2′‐BTPCz.

Compared to the BTPs from our previous studies an electron‐rich carbazole unit was introduced to the parent 2,2′‐BTP^[7e]^ in order to equip the molecules with additional donor‐acceptor interactions orthogonal to the common C−H⋅⋅⋅N bonds dominating the self‐assembly of the BTPs.[^7a^, ^7b^, ^7g^] Details on the synthesis are found in the Supporting Information.

STM was performed at the highly oriented pyrolytic graphite (HOPG)|liquid interface from solutions of 2,2′‐BTPCz in 1,2,4‐trichlorobenzene (TCB) (Figure 2). Highly ordered structures could be observed immediately after the application of the solution and the start of the scanning process, depending on the experimental conditions (quality of the tip and scan location). Fairly large domains in the range of thousands of nm^2^ are visible with a lamellar structure (Figure 2a). Upon closer inspection of the images, different values for the width of the parallel rows can be seen not only in different domains, but also in the same domain (see colored boxes in Figure 2b). Jumps in width and subsequent defects can also be observed within a row (pink oval in Figure 2b).

**Figure 2:**
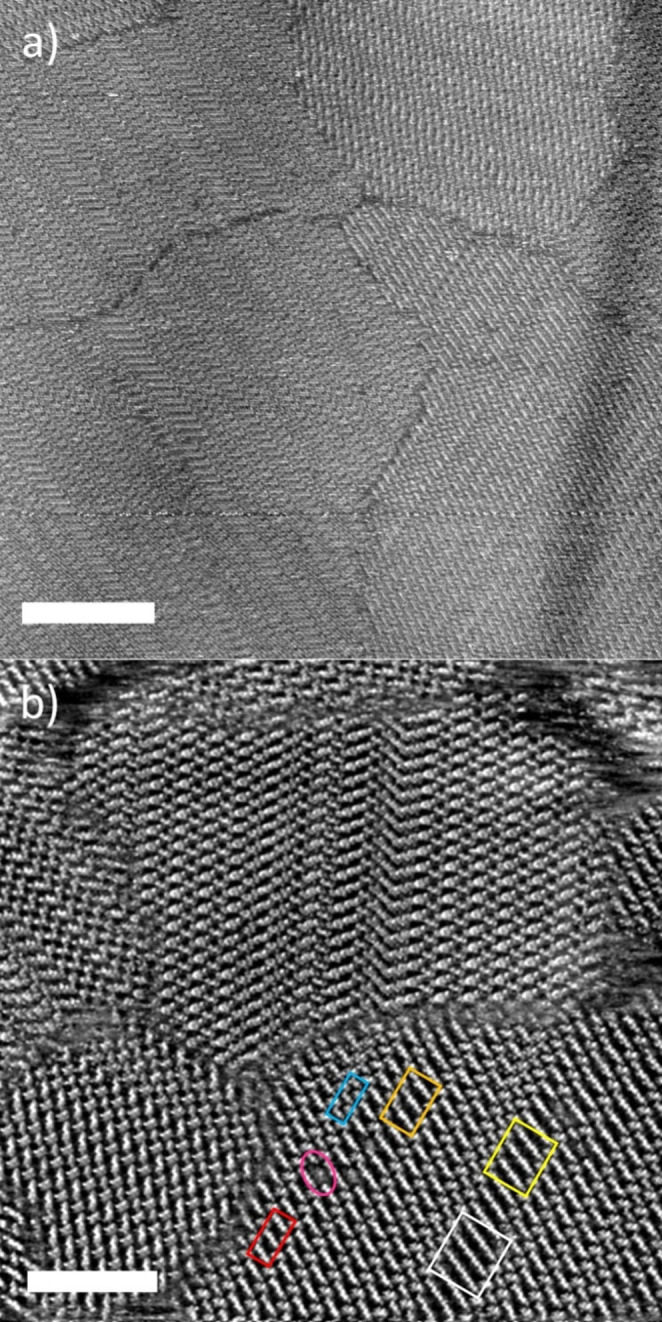
a) Large scale STM image (*c*=1.0 mg mL^−1^, *I*_T_=32 pA, *U*_T_=−460 mV. Scale bar 35 nm. b) Detailed STM image (*c*=1.0 mg mL^−1^, *I*_T_=38 pA, *U*_T_=−328 mV). The boxes show each three sequences of consecutive molecules of configuration B with various sequence lengths *n*_B_ (blue: *n*_B_=1, red: *n*_B_=2, orange: *n*_B_=3, yellow: *n*_B_=4, white: *n*_B_=5), and the pink oval depicts a defect caused by a jump from *n*_B_=1 to *n*_B_=2 within a lamella. Scale bar 20 nm.

High resolution images enable the contrast to be clearly assigned to the arrangement of the molecules in the 2D structures (Figure 3). Here, two main binding motifs can be recognized. Individual molecules interact with neighboring molecules via two weak C−H⋅⋅⋅N hydrogen bonds (yellow molecules (configuration A) in Figure 3a and d) similar to the interactions already described for the parent BTPs.[^7a^, ^7b^] On the other hand, there is a slight π‐π and/or C−H⋅⋅⋅π overlap between carbazole donor and pyridine acceptor units (blue molecules (configuration B) and red moieties in Figure 3a and d, respectively). The latter interactions are responsible for the formation of sequences of molecules with the same configuration B and sequence lengths *n*_B_=1 to 5 (colored boxes in Figure 2b). The structure is confirmed by DFT calculations of the unit cell of a dimer (Figure 3b and c). In order to keep the effort within limits, only the fragments of the peripheral molecules A in vicinity of the molecules B were used for the calculations (see Figure 3d). In addition, a single graphene layer was employed as substrate. The calculations give (C−)H⋅⋅⋅N distances between 2.39 and 2.42 Å, which clearly indicates the presence of (weak) hydrogen bonds.

**Figure 3:**
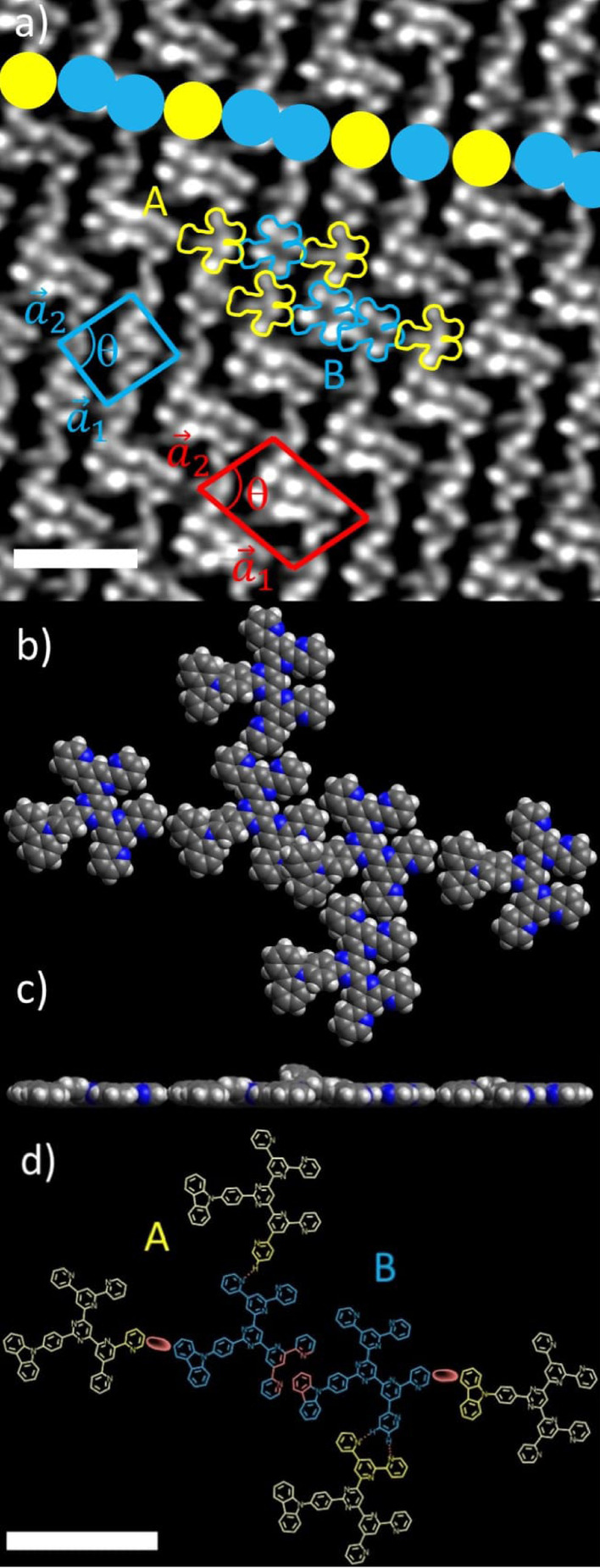
a) More detailed STM image (*c*=0.33 mg mL^−1^, *I*_T_=28 pA, *U*_T_=−730 mV) with overlayed contours of molecules. Yellow: configuration A, blue: configuration B molecules with corresponding unit cells (blue, *n*_B_=1; red, *n*_B_=2). The coloured balls show a supramolecular polymer chain stabilized by vdW interactions and π overlap. Scale bar 4 nm. b)–d) Unit cell of a dimer of B (see red unit cell in a) obtained from DFT calculations, the graphene substrate has been omitted for the sake of clarity. Scale bar 2 nm. b) Top view. c) Side view with protruding carbazole moiety due to π overlap. d) Detailed molecular structure of the unit cell from (b) and (c) with intermolecular interactions within the and between the polymer chains (dashed lines: hydrogen bonds, red ovals: vdW interactions, red aromatic bonds: π overlap). Only the dark coloured parts were used for the calculations and the light yellow substructures were added manually afterwards.

This enables the identification of five oblique unit cells for the monomeric and oligomeric sequences with a constant vector $\left|{\vec{a}}_{2}\right|$ , a systematically increasing $\left|{\vec{a}}_{1}\right|$ and a decreasing angle *θ* with increasing sequence length *n*_B_ (Table 1). For the unit cell of the dimer the theoretical values are in good agreement with the experimental data (see Table 1).

**Table 1:** Parameters of the oblique unit cells for the sequence lengths *n*_B_=1 to 5.

Sequence length *n*_B_	1	2^[a]^	3	4	5
$\left|{\vec{a}}_{1}\right|$ [nm]	2.67± 0.05	4.16± 0.33 (4.1)	5.63± 0.38	7.25± 0.17	9.45± 0.1
$\left|{\vec{a}}_{2}\right|$ [nm]	2.86± 0.04	2.87± 0.04 (2.8)	2.87± 0.07	2.75± 0.11	2.75± 0.1
*θ* [°]	88± 1	81±6 (75)	73± 6	77± 5	74± 1
A^[b]^ [nm^2^]	3.82± 0.24	3.94± 1.33	3.86± 1.89	3.88± 1.69	4.15± 1.30
*ρ* ^[b]^ [nm^−2^]	0.262± 0.016	0.254± 0.086	0.259± 0.127	0.258± 0.112	0.241± 0.075

[a] Values in parentheses from DFT calculations (Figure 3b–d); [b] A: area per molecule, packing density *ρ*=*A* ^*−1*^.

In order to better understand this complex mixture of different structures, we statistically evaluated the distribution of the sequence lengths *n*_B_ (Table S1). Please note that configuration A (yellow molecules in Figure 3) is never directly adjacent to another A molecule, i.e., only *n*_A_=1 exists. In addition, STM experiments were carried out at three different concentrations *c* (0.33, 1.0, 3.3 mg mL^−1^) (Figure S2 to S4). For the highest *c* the sequence fractions *N*_B_ *(n)* are strictly decreasing with increasing *n*_B_ (Figure 4). The situation changes significantly for decreasing *c*. A marked increase in *N*_B_(2) at the expense of *N*_B_(1) is seen. Sequence length distributions typically occur in copolymer chains that are formed through a (free radical) chain growth mechanism. The distributions depend on the relative reactivities *r* of the monomers with the chain ends and the feed ratio *f* of the monomers. In this purely kinetic approach, the terminal model is mostly used, in which only the very last repeating unit at the chain end is considered. This leads to a strict decrease of the fraction of sequence length *N*_B_(*n*) with increasing *n*. Thus, this model might explain the black curve in Figure 4 for the highest *c* but will definitely fail for lower *c*. If you look not only at the very last but also the penultimate unit at the chain end, the behaviour changes fundamentally. Four copolymerization parameters *r*_A_, *r*_A_′, *r*_B_, and *r*_B_′ are defined for this penultimate model (see Scheme S2). We consider the two configurations A and B in Figure 3 as two comonomers due to their specific binding to neighboring molecules and assume *r*_A_ and *r*_A_′=0 (no “reaction” of A with A, see above) and *f*_A_=*f*_B_=0.5 (both configurations are equally likely). With these assumptions, the experimental values of *N*_B_(*n*) can be fitted excellently with the penultimate model (open symbols in Figure 4) and are all within the error margins. Details on the simulation can be found in Supporting Information and in the literature.^[8]^ The resulting two parameters *r*_B_ and *r*_B_′ are clearly distinguishable and show a significant development with decreasing concentration (Figure 5). While at high *c* both are below 1 and are quite similar, *r*_B_ continues to decrease slightly and *r*_B_′ increases sharply with decreasing *c*. This means that at high *c* the system can be described satisfactorily by the terminal model, but at low *c* the effect of the penultimate unit must not be neglected. In an alternative evaluation of the sequence distribution, the fractions of rows with a certain sequence length were determined (Figure S5 and S6 and Table S1). While the exact numbers are slightly different especially for the medium concentration compared to Figure 4—the differences result from the different lengths of the rows and the relatively small absolute numbers of rows—the trend of the concentration dependence within the experimental error is the same. The similar results of both evaluation methods indicate a similar mechanism for generating single sequences and many identical sequences within a row (see below). A very good agreement between the experimental and the simulated values can also be found for the incorporated quantities of configuration A and B, *F*_A_ and *F*_B_, and the mean sequence lengths *n*_B_(*av*) (Figure S7).

**Figure 4:**
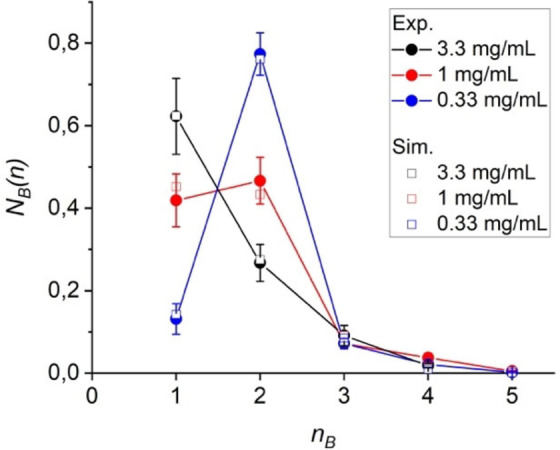
Number fractions *N_B_* (*n*) of the sequence lengths *n*_B_ of configuration B (blue in Figure 3) depending on concentration *c*. The open symbols are fits from the copolymerization equations of the penultimate model; the vertical bars represent the experimental errors.

**Figure 5:**
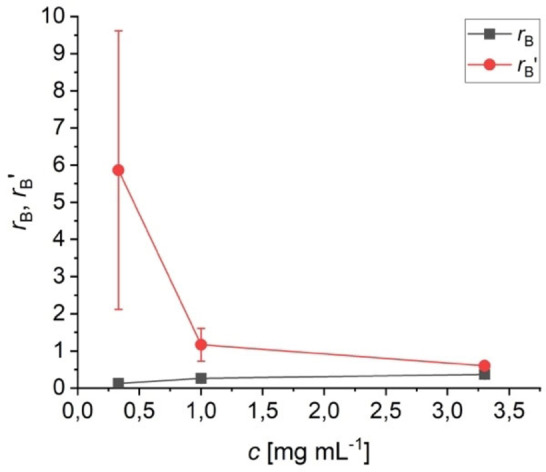
Reactivity ratios *r*_B_ and *r*_B_′ depending on concentration *c*. They were derived from the simulation of the sequence length distribution (Figure 4) according to the penultimate model.

The excellent fit of the penultimate model to the experimental data suggests the formation of the supramolecular structure through a kinetically driven copolymerization mechanism. This raises the question whether the supramolecular polymerization has already taken place in solution or only on the substrate. A counterintuitive effect of concentration on 2D structure formation described in the literature is attributed to pre‐aggregation in solution.^[9]^ The formation of polymers in solution can be ruled out because ^1^H NMR and UV/Vis spectroscopy as well as DLS (Dynamic Light Scattering) measurements are independent on the concentration or only prove the presence of monomers (Figure S8–S10). Thus, the polymers must have been formed on the substrate.

The challenges in applying the copolymerization model to the self‐assembly process on the graphite are i) the combination of a 1D mechanism with a 2D system, ii) the marked excess of dimeric sequences, and iii) the significant concentration dependence of the sequence length distribution. In order to answer these questions and finally to derive a plausible mechanism we determined the energy values from the DFT calculations of the optimized structure in Figure 3b–d. In the absence of the graphene layer exothermic values around −6 kJ mol^−1^ (vdW), −20 kJ mol^−1^ (single H bond), and −29 kJ mol^−1^ (double H bond) were obtained. In order to determine the value for the π overlap, the graphene was included in the calculation and an endothermic value of +22 kJ mol^−1^ was obtained for the overlap. However, it must be taken into account that this endothermic process is accompanied by a gain in adsorption energy due to the smaller area required by the overlapping molecules. This corresponds to a lateral shift of approximately 0.4 nm per π overlap and ultimately leads to a total gain of around −18 kJ mol^−1^ (for details see Supporting Information). In addition, it is expected that the differences between the energy values in solution will be much smaller than in the gas phase due to the structural similarity of the molecules of the solvent and solute. Thus, the small differences facilitate the kinetic vs. the thermodynamic control of the self‐assembly process, which supports the proposed copolymerization model. Since the copolymerization parameters represent ratios of rate constants, the dependence of r on the concentration *c* (see Figure 5) can be expressed by the differences of the apparent activation energies ${\Delta \Delta G}^{\#}$ according to equations S1 and S2. At high *c* (3.3 mg mL^−1^), ${\Delta \Delta G}_{B}^{\#}$ (trimer over dimer formation) and ${\Delta \Delta G}_{B}^{\#}{}^{'}$ (dimer over monomer formation) are extremely low and almost identical with +2 and +1 kJ mol^−1^ at room temperature. This is consistent with the negligible differentiation between the penultimate units. The corresponding values for low *c* (0.33 mg mL^−1^) are +5 and −4 kJ mol^−1^, which explains the kinetically preferred formation of dimers over monomers. Although ${\Delta \Delta G}^{\#}$ should be concentration independent, the complex mechanism of the supramolecular polymerization could be concentration dependent. In addition, the overall slower kinetics at lower concentrations could increasingly support the thermodynamic control, since the π overlap is thermodynamically favored (see above). However, the overall structure is kinetically determined even at low *c*; otherwise, almost exclusively supramolecular polymers should be formed instead of oligomers. Kinetic control is also supported by DFT calculations of a trimer on a large graphene layer with two π overlaps. The energy minimization leads to a local minimum with a dimer (one π overlap) and a vdW‐bound molecule (see Figure S11). Apparently, the activation energy for a lateral movement of a planar adsorbed BTP molecule is too high to reach the global minimum of three planar adsorbed molecules.

From the findings above, we propose a mechanism that corresponds to the generally accepted nucleation and growth model for crystallization in both 2D and 3D. For the present system, however, we go into more detail and describe it for the combination of two processes (Scheme 1): i) kinetically controlled nucleation and growth (=genesis of a certain sequence of length *n*_B_ in the direction of a polymer chain (linked by vdW interactions and π overlap, see colored dots in Figure 3a) and, thus, nucleation of a new row) and ii) thermodynamically controlled growth orthogonal to a sequence (=growth along a row, linked by H bonds). The latter guarantees the high order within a row, i.e., high crystallinity with only rare defects.

**Scheme 1:**
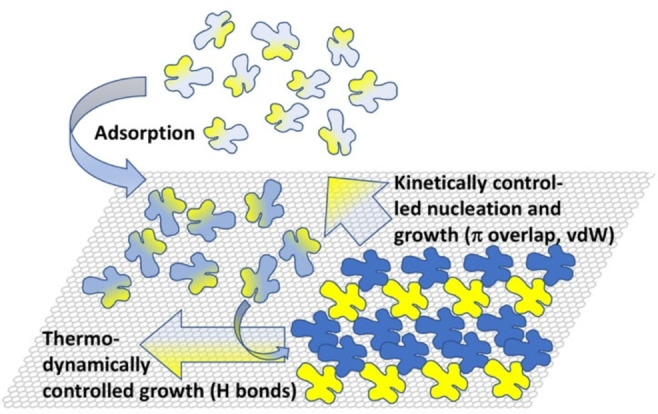
Kinetically and thermodynamically driven mechanism of 2D self‐assembly of oligopyridines to copolymers with a distribution of their sequences on graphite. The straight arrows indicate the growth direction along and orthogonal to the copolymer chains, respectively.

The statistical approach, in which the 1D copolymer growth shows a distribution of the sequence lengths, applies in general to all types of kinetically controlled copolymers (alternating, block, random etc.). We believe it can also be applied to the generation of 2D (co)polymers and used as a guide for kinetic control. Different sequence lengths do not necessarily have to be present in one domain but can be distributed in different domains. This depends on the number of nucleation events per domain. In future mechanistic studies of related solvent‐based systems, it is strongly recommended to consider the influence of kinetics and their quantitative evaluation.

## Conflict of interest

The authors declare no conflict of interest.

## Supporting information

As a service to our authors and readers, this journal provides supporting information supplied by the authors. Such materials are peer reviewed and may be re‐organized for online delivery, but are not copy‐edited or typeset. Technical support issues arising from supporting information (other than missing files) should be addressed to the authors.

Supporting Information

## Data Availability

The data that support the findings of this study are available in the Supporting Information of this article.
