# Supplementary material for: Static Scanning Tunneling Microscopy Images Reveal the Mechanism of Supramolecular Polymerization of an Oligopyridine on Graphite
Source: Angew Chem Int Ed Engl. 2022 Feb 21;61(16):e202117580. doi: 10.1002/anie.202117580 (PMC9307023; doi:10.1002/anie.202117580)
Supplement: Supplementary file 1 — Supporting Information [file ANIE-61-0-s001.pdf]

Angewandte Chemie
Eine Zeitschrift der Gesellschaft Deutscher Chemiker

Supporting Information

Static Scanning Tunneling Microscopy Images Reveal the Mechanism of Supramolecular Polymerization of an Oligopyridine on Graphite

*F. D. Goll, G. Taubmann, U. Ziener**

Supporting Information

Content

1 SYNTHESIS 2

2 DLS 3

3 COPOLYMERIZATION EQUATIONS ACCORDING TO THE PENULTIMATE MODEL 4

4 STM IMAGES AND STATISTICAL EVALUATION 5

4.1 STM IMAGES 5

4.2 SIMULATION OF THE NUMBER FRACTIONS $N_B(N)$ AND DETERMINATION OF THE COPOLYMERIZATION PARAMETERS R 7

5 ¹H NMR STUDIES 8

6 UV/VIS STUDIES 10

7 CALCULATIONS 10

7.1 STRUCTURE OPTIMIZATION 10

7.2 ESTIMATION OF ADSORPTION ENERGY 11

8 REFERENCES 11

1 Synthesis

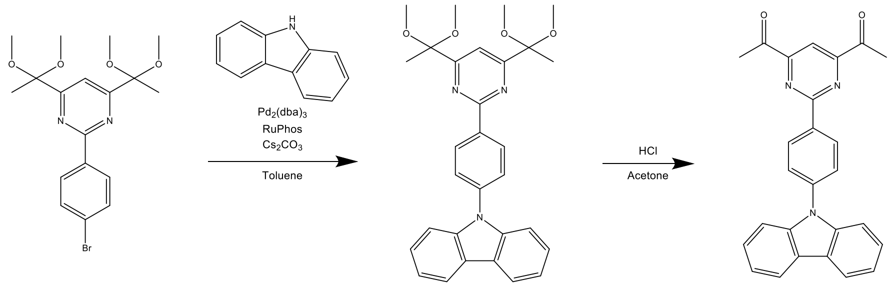

1 2 3

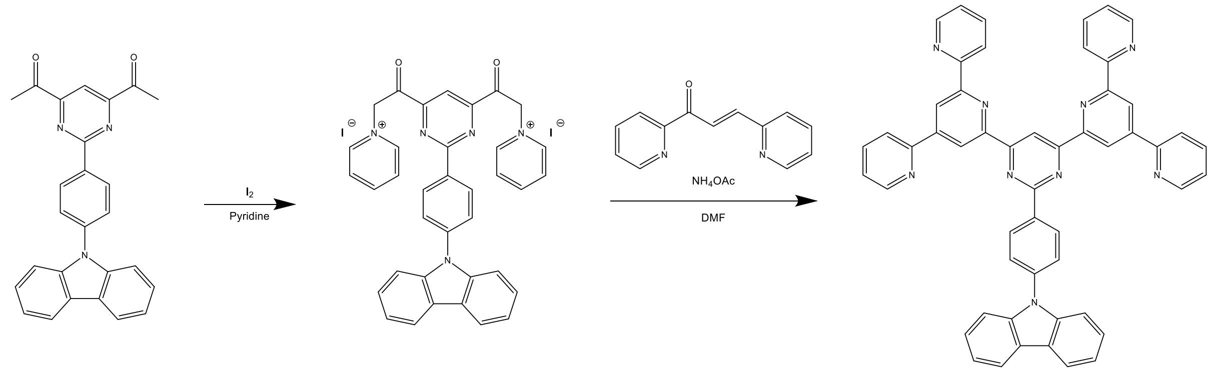

3 4 5

Scheme S 1. Synthesis scheme for 2,2'-BTPCz.

General. Protected diacetylpyrimidine **1**[1] and 1,3-di-(2'-pyridyl)-2-propen-1-one[2] were synthesized according to literature procedures. Cesium carbonate, carbazole, dimethylformamide, pyridine, RuPhos, toluene, and tris(dibenzylideneacetone)dipalladium(0) were purchased from Sigma Aldrich and used without further purification. Acetone, ammonium acetate, hydrochloric acid and iodine were obtained from VWR Chemicals and used as received. The NMR data were obtained on a Bruker DRX 500 at 400.1 MHz (^{1}H) and 100.6 MHz (^{13}C), respectively. Chemical shifts are given in ppm relative to $CDCl_3$ (^{1}H = 7.26 ppm, ^{13}C = 77.2 ppm), $C_2D_2Cl_4$ (^{13}C = 74.4 ppm) or DMSO (^{1}H = 2.50 ppm). Mass spectrometry was done using a Bruker solariX for MALDI and ESI measurements and a Thermo Scientific ISQ LT for EI/CI measurements.

9-(4-(4,6-bis(1,1-dimethoxyethyl)pyrimidin-2-yl)phenyl)-9H-carbazole **2**. Compound **1** (411 mg, 1 mmol), carbazole (176 mg, 1.05 mmol), cesium carbonate (489 mg, 1,5 mmol), RuPhos (19 mg, 40 µmol) and Pd$_2$(dba)$_3$ (18 mg, 20 µmol) were dissolved in 20 mL dry toluene and the solution was degassed. The mixture was refluxed until the reactants were consumed according to TLC ($CHCl_3$, silica). The solution was cooled down and filtered through a celite plug. The solvent was removed and the residue dried in vacuo, yielding a brown solid. Yield 396 mg (80%). ^{1}H NMR ($CDCl_3$, 400.1 MHz, 295 K): δ 8.83-8.81 (dd, 2H, J = 4.7, 2 Hz), 8.17-8.15 (d, 2H, J = 7.5 Hz), 7.90 (s, 1H), 7.71-7.69 (dd, 2H, J = 4.7, 2.0 Hz), 7.51-7.49 (d, 2H, J = 8.1 Hz), 7.45-7.41 (td, 2H, J = 6.4, 1.2 Hz), 7.33-7.29 (td, 2H, J = 6.4, 1.0 Hz), 3.29 (s, 12H), 1.76 (s, 6H). ^{13}C NMR (126 MHz, $CDCl_3$): δ 169.90, 163.66, 140.72,140.03, 136.93, 130.48, 126.95, 126.55, 123.70,120.72, 120.63, 114.31, 110.27, 101.72, 49.77, 23.55. MS (CI): calculated m/z 497.2 (M$^+$); found 497.2.

1,1'-(2-(4-(9H-carbazol-9-yl)phenyl)pyrimidine-4,6-diyl)bis(ethan-1-one) **3**. Compound **2** (390 mg, 784 µmol) was deprotected by dissolving it in 25 mL of acetone and adding concentrated aqueous hydrochloric acid (2.5 mL, 28.5 mmol). A yellow precipitate was immediately formed. The dispersion was stirred for 1 h at room temperature. Subsequently, the precipitate was filtered off and washed with a small amount of cold acetone. The yellow solids were dried in vacuo. Yield 317 mg (quant.). ^{1}H NMR ($CDCl_3$, 400.1 MHz, 295 K): δ 8.78-8.76 (d, 2H, J = 8.5 Hz), 8.21 (s, 1H), 8.11-8.09 (d, 2H, J = 7.7 Hz), 7.75-7.73 (d, 2H, J = 8.5 Hz), 7.51-7.49 (d, 2H,

J = 8.2 Hz), 7.42-7.38 (t, 2H, J = 7.7 Hz), 7.29-7.25 (t, 2H, J = 7.4 Hz), 2.81 (s, 6H). ^{13}C NMR (126 MHz, $CDCl_3$): δ 199.55, 164.68, 161.62, 141.00, 140.53, 134,98, 130.48, 127.06, 126.60, 123.80, 120.85, 120.77, 111.10, 110.29, 26.23. MS (CI): calculated m/z 405.1 (M^+); found 405.1.

1,1'-((2-(4-(9H-carbazol-9-yl)phenyl)pyrimidine-4,6-diyl)bis(2-oxoethane-2,1-diyl))bis(pyridin-1-ium) iodide ***4***. Compound 3 (310 mg, 765 µmol) and iodine (391 mg, 1.54 mmol) were dissolved in 13 mL dry pyridine and refluxed for 5 h. A yellow precipitate formed after half an hour. The mixture was subsequently cooled down and the residue was separated using a centrifuge. The solid was washed three times with isopropyl alcohol and then dried in vacuo. Yield 539 mg (86%). ^{1}H NMR (DMSO, 400.1 MHz, 295 K): δ 9.05-9.03 (d, 4H, *J* = 5.5 Hz), 8.82-8.80 (d, 2H, *J* = 8.2 Hz), 8.73-8.72 (d, 2H, *J* = 4.6 Hz), 8.37-8.35 (d, 4H, *J* = 7.6 Hz), 8.21 (s, 1H), 8.08-8.06 (d, 2H, *J* = 8.5 Hz), 7.69-7.67 (d, 2H, *J* = 8.0 Hz), 7.56-7.55 (d, 2H, *J* = 7.5 Hz), 7.53-7.51 (d, 2H, *J* = 7.5 Hz), 7.39-7.37 (d, 2H, *J* = 7.0 Hz), 6.77 (s, 4H).

2,2'-BTPCz ***5***. Bispyridinium salt **4** (245 mg, 300 µmol), 2,2'-diazachalcone (126 mg, 600 µmol), and dry ammonium acetate (1.156 g, 15 mmol) were dissolved in 10 mL dry DMF. The mixture was degassed and stirred at 65 °C under argon. After no more chalcone could be detected by TLC (MeOH/EtOAc 1:4, silica), the reaction was terminated. After cooling to room temperature, the precipitate was separated using a centrifuge and washed twice with methanol. The light brown solids were dried in vacuo. Yield 51 mg (22%). ^{1}H NMR ($CDCl_3$, 400.1 MHz, 295 K): δ 9.84 (s, 1H), 9.44 (d, 2H, *J* = 1.6 Hz), 9.26 (d, 2H, *J* = 1.7 Hz), 9.15- 9.04 (m, 2H), 8.93 (d, 2H, *J* = 7.8 Hz), 8.88 (dd, 2H, *J* = 4.4, 1.5 Hz), 8.84- 8.77 (m, 2H), 8.19 (d, 2H, *J* = 7.7 Hz), 8.16 (d, 2H, *J* = 8.0 Hz), 8.01-7.89 (m, 4H), 7.88 – 7.81 (m, 2H), 7.61 (d, 2H, *J* = 8.2 Hz), 7.51-7.39 (m, 6H), 7.37-7.30 (m, 2H). ^{13}C NMR (126 MHz, $C_2D_2Cl_4$): δ 165.07, 164.22, 157.24, 156.58, 155.59, 155.18, 150.65, 149.79, 149.57, 141.46, 140.60, 137.61, 137.39, 137.02, 130.77, 127.37, 126.62, 124.42, 124.28, 124.21, 121.90, 121.80, 120.86, 120.76, 119.83, 112.97, 110.59. MS (MALDI-FTICR): calculated m/z 784.2937 ($M+H^+$); found 784.2906.

2 DLS

All DLS measurements were conducted using a Malvern Panalytical Zetasizer Nano ZS. Several measurements with different concentrations of 2,2'-BTPCz in 1,2,4-trichlorobenzene were performed to exclude possible aggregate formation. The curves in Figure S 2 display the average of several measurements. It can be seen that the mean size is about 0.7 nm for both concentrations, which indicates that there are no aggregates present. Although sub-nanometre sized particles are at the limits of the instrument's performance, the absence of readings above 1 nm means that the formation of aggregates can be excluded.

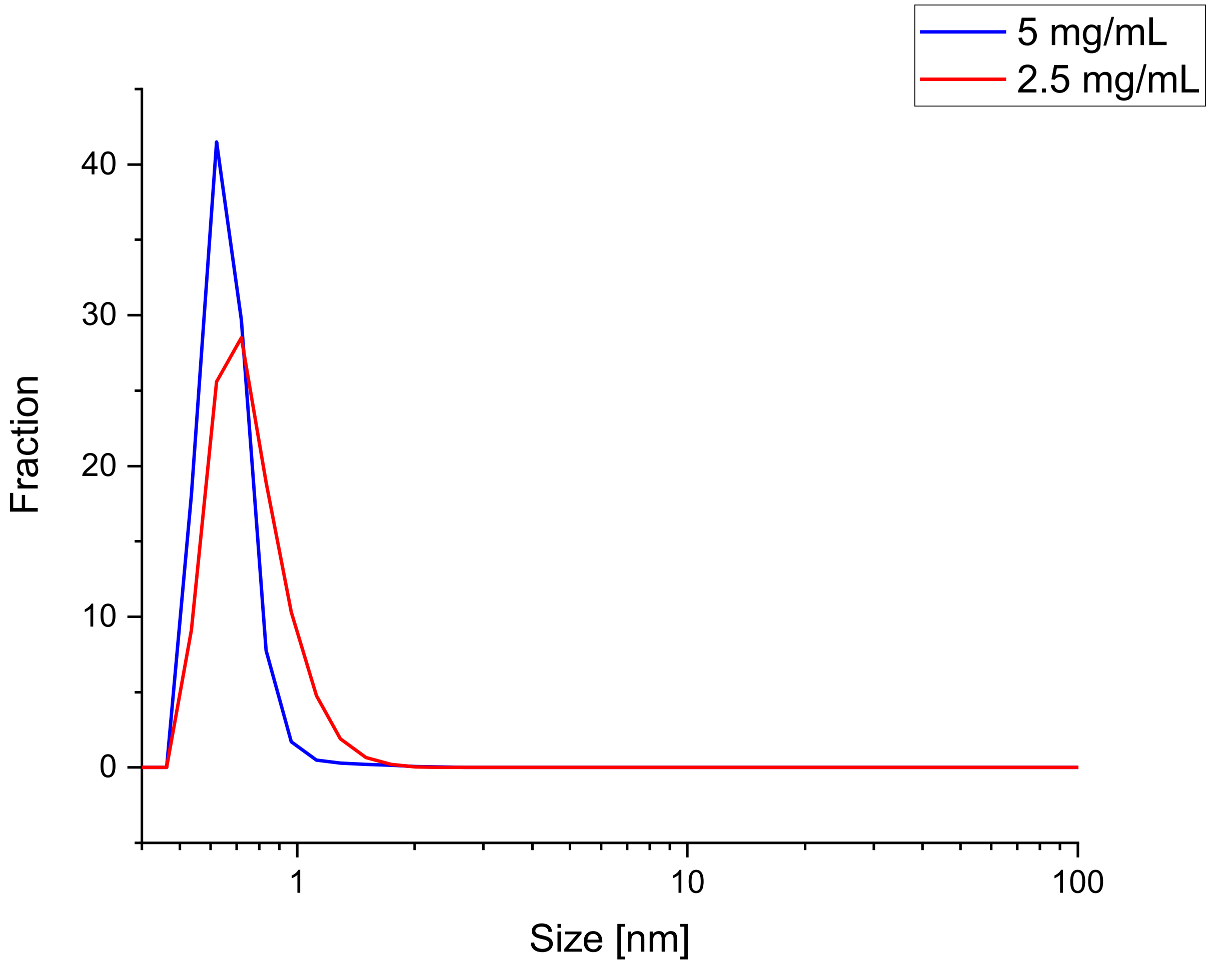

Figure S 1. Size number mean curves of 2,2'-BTPCz in TCB at different concentrations.

3 Copolymerization equations according to the Penultimate Model

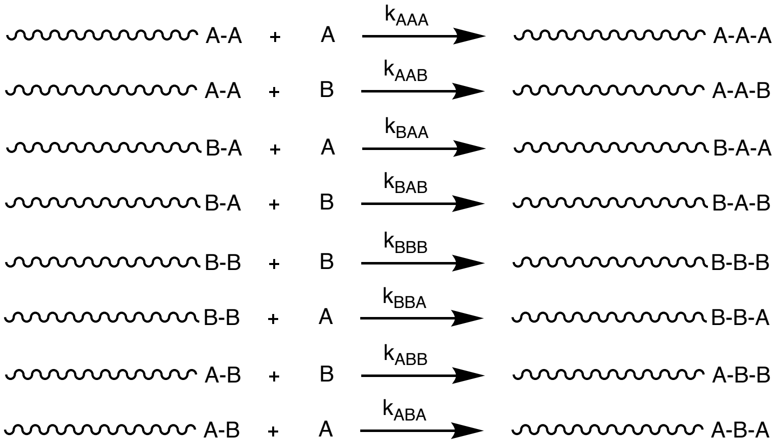

$$r_A = \frac{k_{AAA}}{k_{AAB}}$$

$$r_A' = \frac{k_{BAA}}{k_{BAB}}$$

$$r_B = \frac{k_{BBB}}{k_{BBA}}$$

$$r_B' = \frac{k_{ABB}}{k_{ABA}}$$

Scheme S 2. Chain growth polymerization of two monomers with penultimate effect and definition of the copolymerization parameters r_A, r_A', r_B, and r_B'.

The classic Arrhenius approach delivers the following equations assuming that the pre-exponential factors are equal since there is only one type of reactants:

$$r_B = \frac{k_{BBB}}{k_{BBA}} = \frac{A_{BBB}}{A_{BBA}} * e^{-\frac{\Delta G^{\#}_{BBB} - \Delta G^{\#}_{BBA}}{RT}} \Rightarrow \Delta\Delta G^{\#}_{B} = -RT\ln(r_B) \qquad \text{Eq. S1}$$

$$r_B' = \frac{k_{ABB}}{k_{ABA}} = \frac{A_{ABB}}{A_{ABA}} * e^{-\frac{\Delta G^{\#}_{ABB} - \Delta G^{\#}_{ABA}}{RT}} \Rightarrow \Delta\Delta G^{\#\prime}_{B} = -RT\ln(r_B') \qquad \text{Eq. S2}$$

4 STM images and statistical evaluation

4.1 STM images

Between 1800 and 7800 monomeric and oligomeric sequences of configuration B (between 2500 and 11500 molecules) were counted manually from minimum three images or domains for each concentration (Figure S 2 – Figure S 4). Contrasts in the images which could not unambiguously assigned to a specific sequence were omitted. The corresponding numbers are shown in Table S 1. The errors were determined from the number fractions of the sequences in every image or domain, respectively. In addition, the rows with the respective sequences were counted independently. If a jump from one sequence to another occurred within a row, each sequence block was counted as a separate row.

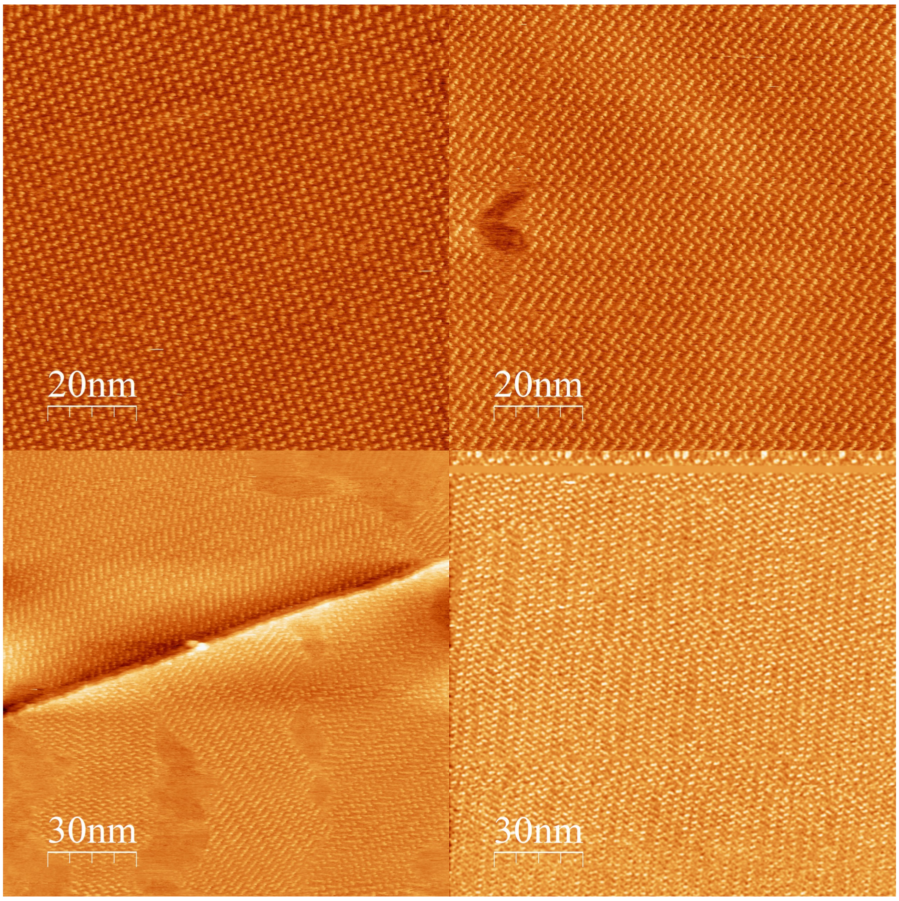

Figure S 2. STM images at different locations deposited from TCB (c = 0.33 mg mL^{-1}).

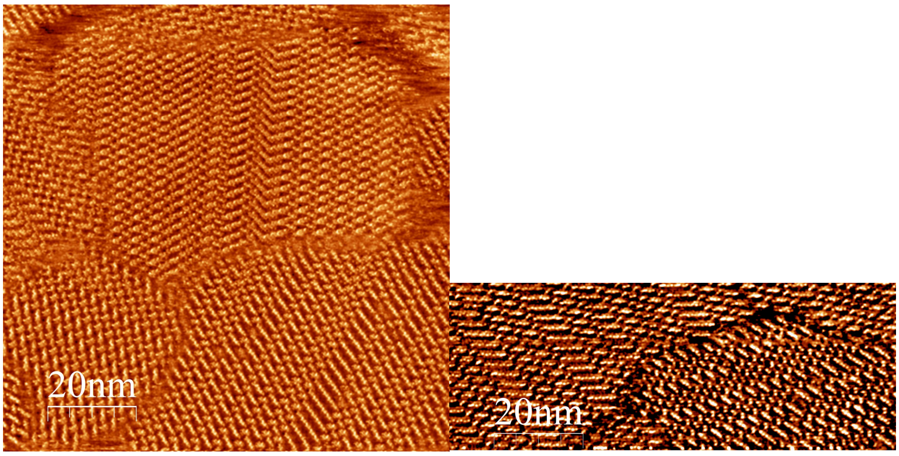

Figure S 3. STM images at different locations deposited from TCB (c = 1.0 mg mL^{-1}).

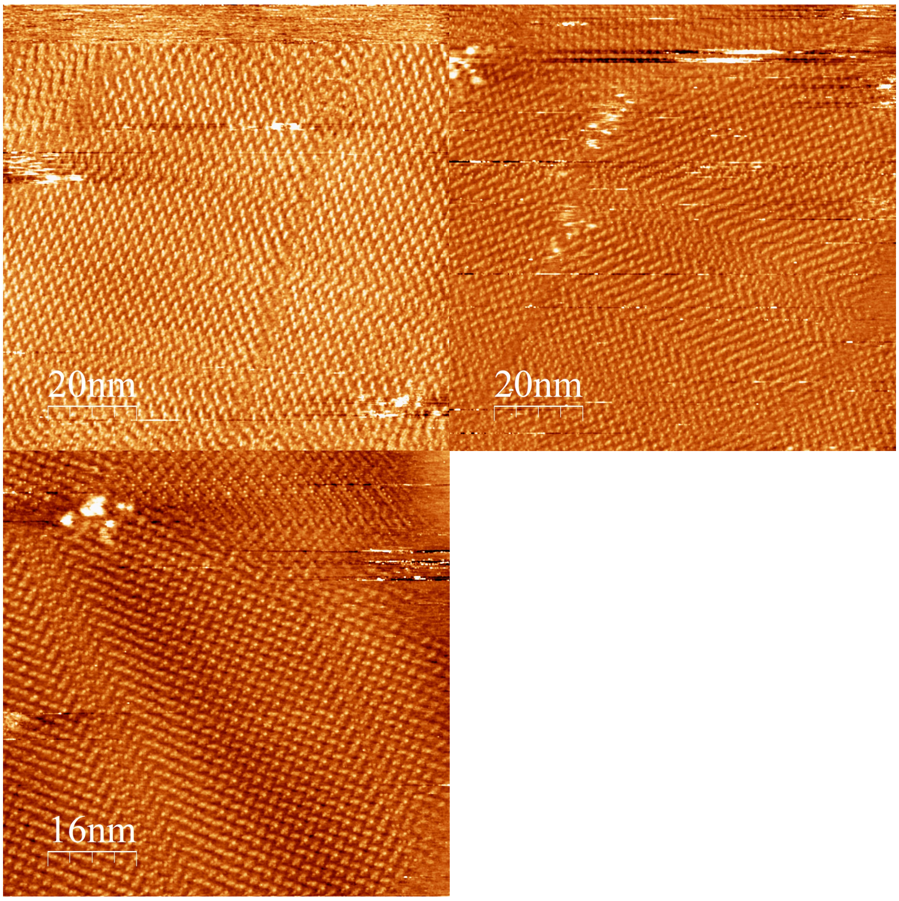

Figure S 4. STM images at different locations deposited from TCB (c = 3.3 mg mL^{-1}).

Table S 1. Frequencies of monomeric and oligomeric sequences of configuration B at different concentrations determined from Figure S 2 – Figure S 4.

		0.33 mg mL^{-1}		1 mg mL^{-1}		3.3 mg mL^{-1}	
	Sequence length n_B	N[a]	$N_B(n)$[a]	N[a]	$N_B(n)$ [a]	N[a]	$N_B(n)$[a]
	1	419	0.131±0.037	413	0.419±0.064	635	0.622±0.092
	2	2527	0.774±0.051	460	0.467±0.056	273	0.268±0.045
Total	3	235	0.072±0.012	71	0.072±0.013	93	0.091±0.025
sequences	4	68	0.021±0.008	37	0.038±0.011	19	0.019±0.005
	5	7	0.002±0.0009	5	0.005±0.002	0	0

	$\sum N$	3266		986		1020	
	n_B(av)[b]	1.99		1.74		1.51	
	r_B[c]	0.13		0.26		0.37	
	r_B‘[c]	6.02		1.21		0.60	
	1	21	0.174±0.051	19	0.202±0.019	58	0.707±0.129
	2	80	0.661±0.165	57	0.606±0.064	19	0.232±0.048
Rows	3	14	0.116±0.031	13	0.138±0.025	4	0.049±0.012
with	4	5	0.041±0.014	4	0.043±0.012	1	0.012±0.005
sequences	5	1	0.008±0.004	1	0.011±0.004	0	0
	$\sum N$	121		94		82	
	n_B(av)[b]	2.05		2.05		1.37	
	r_B[c]	0.32		0.34		0.29	
	r_B‘[c]	3.51		3.94		0.42	

[a] N: number of B sequences of length n_B or number of rows with B sequences of length n_B, respectively, $N_B(n)$: number fraction of n_B; [b] number average sequence length n_B(av); [c] r_B and r_B‘ were determined from Eq. 1 and 2 (see Chapter 4.2)

4.2 Simulation of the number fractions N_B(n) and determination of the copolymerization parameters r

For the determination of the copolymerization parameters r_B and r_B' according to the penultimate model the following equations according to the literature were applied.[3]

$$N_B(n) = \frac{r_B'}{1+r_B'} * \frac{1}{1+r_B} * \left(\frac{r_B}{1+r_B}\right)^{n-2} \quad \text{for } n \geq 2 \quad \text{(Eq. 1)}$$

$$N_B(n) = \frac{1}{1+r_B'} \quad \text{for } n = 1 \quad \text{(Eq. 2)}$$

A fit procedure of the experimental data for $n \geq 2$ with Eq. 1 and r_B and r_B' as fitting parameters was carried out with Origin.[4] r_B' was also determined from Eq. 2. For the simulation of $N_B(n)$ (see Figure 4) the weighted number average of r_B' from Eq. 1 and 2 was taken.

The incorporated amounts of A and B in the copolymer F_A and F_B and the average sequence lengths n_B*(av)* were calculated according to Eq. 3 to 5 with the help of an Excel program provided by the literature.[3] Please note that these simplified equations are only valid for equal feed ratios f_A = f_B = 0.5 (equal probability of both configurations A and B) and r_A = r_A‘ = 0 (no “reaction” between chain end A and monomer A, see Scheme S 2).

$$F_B = \frac{1+2r_B'+r_Br_B'}{2+3r_B'+r_Br_B'} \quad \text{(Eq. 3)}$$

$$F_A = 1 - F_B \quad \text{(Eq. 4)}$$

$$n_B(av) = 1 + r_B'\frac{1+r_B}{1+r_B'} \quad \text{(Eq. 5)}$$

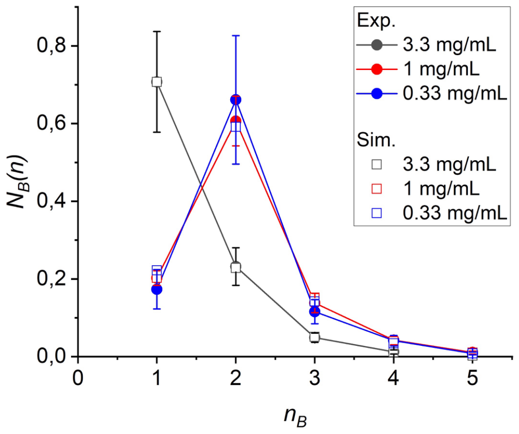

Figure S 5. Number fractions $N_B(n)$ of the rows with sequence length n_B of configuration B depending on concentration c. The open symbols are fits from the copolymerization equations of the penultimate model (Eq. 1 and 2); the vertical bars represent the experimental errors.

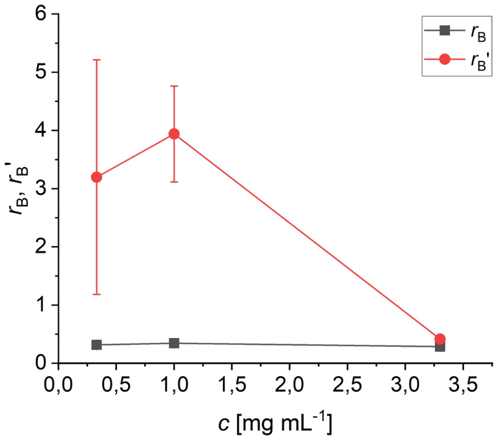

Figure S 6. Reactivity ratios r_B and r_B' depending on concentration c. They were derived from the simulation of the sequence length distribution based on the number fraction of rows with the respective sequence length according to the penultimate model.

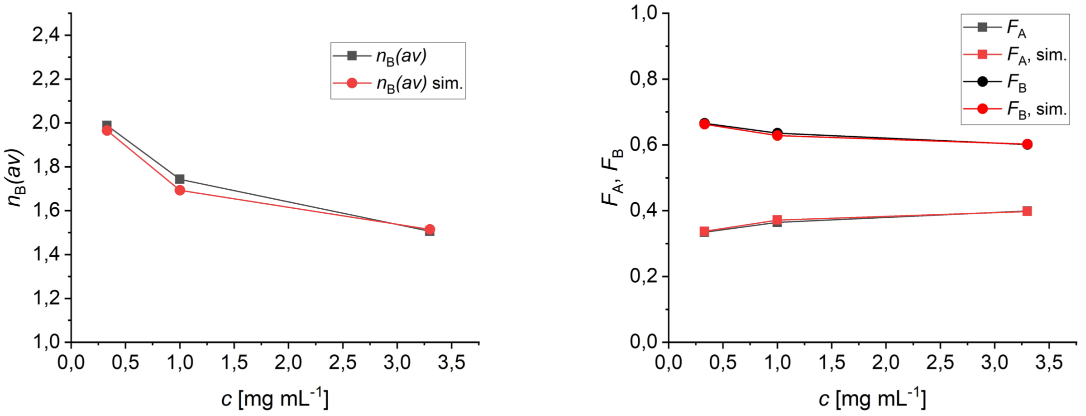

Figure S 7. Experimental (from individual sequences) and simulated average sequence lengths $n_B(av)$ of monomer B (left) and incorporated fractions of monomer A and B, F_A and F_B, (right) depending on concentration.

5 ^{1}H NMR studies

^{1}H NMR spectra of 2,2'-BTPCz in 1,2-dichlorobenzene-d_4 at various concentrations were taken at rt. The peaks were assigned to the respective protons and the chemical shifts show a linear

dependence from the concentration (Figure S 8). Figure S 9 displays the slopes of the straight lines for the different peaks.

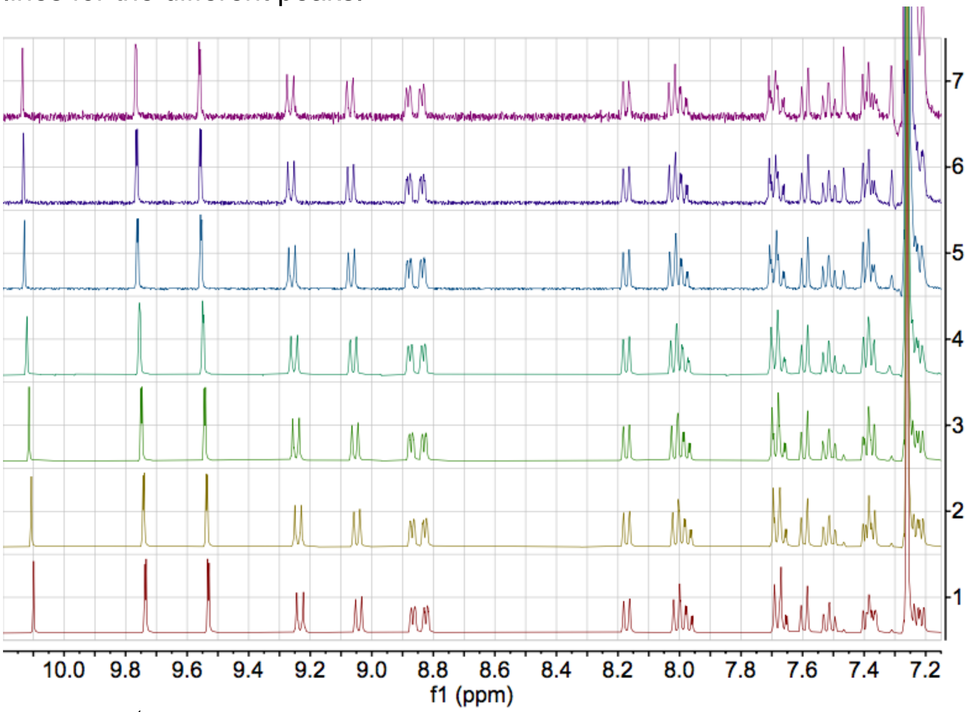

Figure S 8. *^{1}H NMR spectra of 2,2'-BTPCz in 1,2-dichlorobenzene-d_4 at rt and various concentrations (1: 3 mg mL^{-1}, 2: 2.4 mg mL^{-1}, 3: 1.8 mg mL^{-1}, 4: 1.2 mg mL^{-1}, 5: 0.6 mg mL^{-1}, 6: 0.3 mg mL^{-1}, 7: 0.15 mg mL^{-1}).*

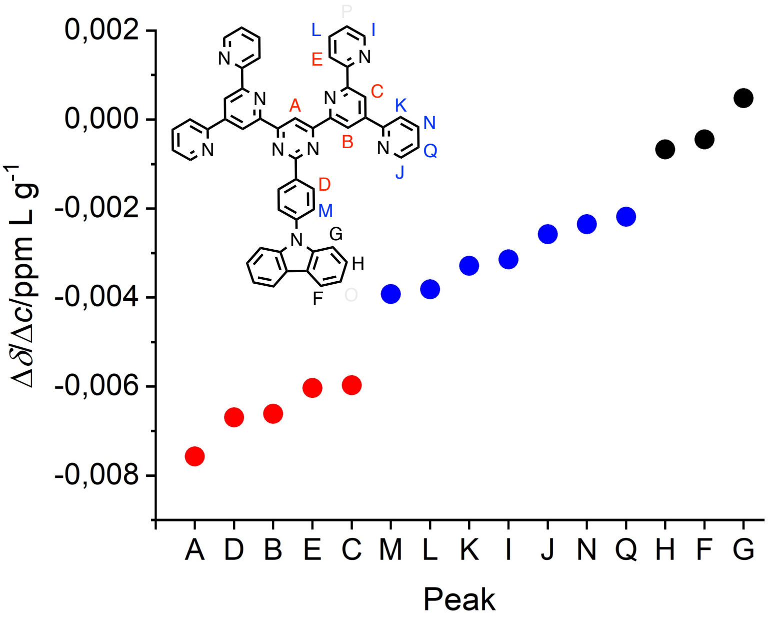

Figure S 9. Change of chemical shift with concentration for the respective peaks measured in 1,2-dichlorobenzene-d_4 at rt. For the missing letters (O, P) no clear assignment could be made. The colors show three groups of peaks with similar shift changes.

6 UV/Vis studies

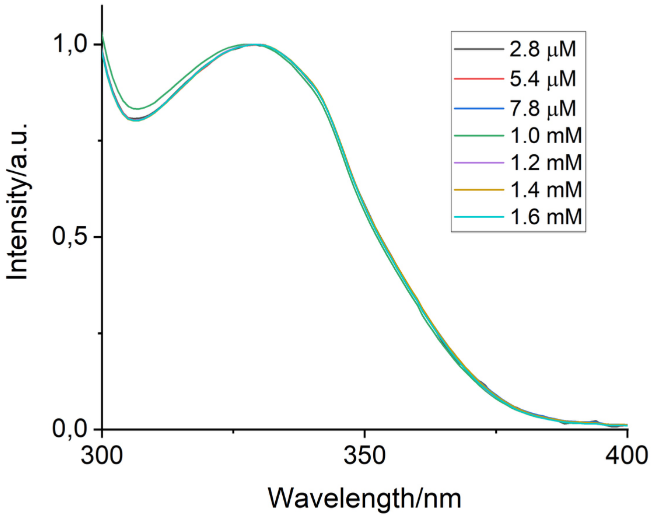

Figure S 10. UV/Vis spectra of 2,2'-BTPCz in TCB at rt with various concentrations (2.2 – 1.3 mg mL^{-1}).

7 Calculations

7.1 Structure optimization

Computational details

The calculations were carried out using the xTB program package (6.4.0) from Grimme's group.[5] The xTB GFN code uses an extended semiempirical tight-binding model parameterized to obtain good values for geometry, forces and nonbonding interactions. The energies were calculated with GFN2-xTB.[6] Some preoptimizations were carried out using the gfn-ff force field provided by recent versions of xTB.[7]

A single graphene layer consisting of 592 C-atoms terminated with 74 H-atoms (26 x 10 C_6-rings) was used as substrate. The graphene layer was optimized in advance. The positions of its atoms were constrained during the subsequent calculations. A trimer on the graphene layer with two π overlaps was optimized. In the starting structure the molecule on the right-hand side was adsorbed in a planar way, while in the two others the carbazole unit was twisted in order to allow for π overlaps. During optimization the left molecule slips from the middle molecule and adsorbs in a planar way on the substrate whereas the middle molecule still stays overlapping with the right one (Figure S 11). In order to achieve a planar structure for this middle molecule, one of the two molecules situated to the left or to the right from it would have to move by a considerable distance. Apparently, the lateral shift of a molecule adsorbed fully on the graphene has a high activation energy. This supports the dominating kinetic control of the self-assembled structures.

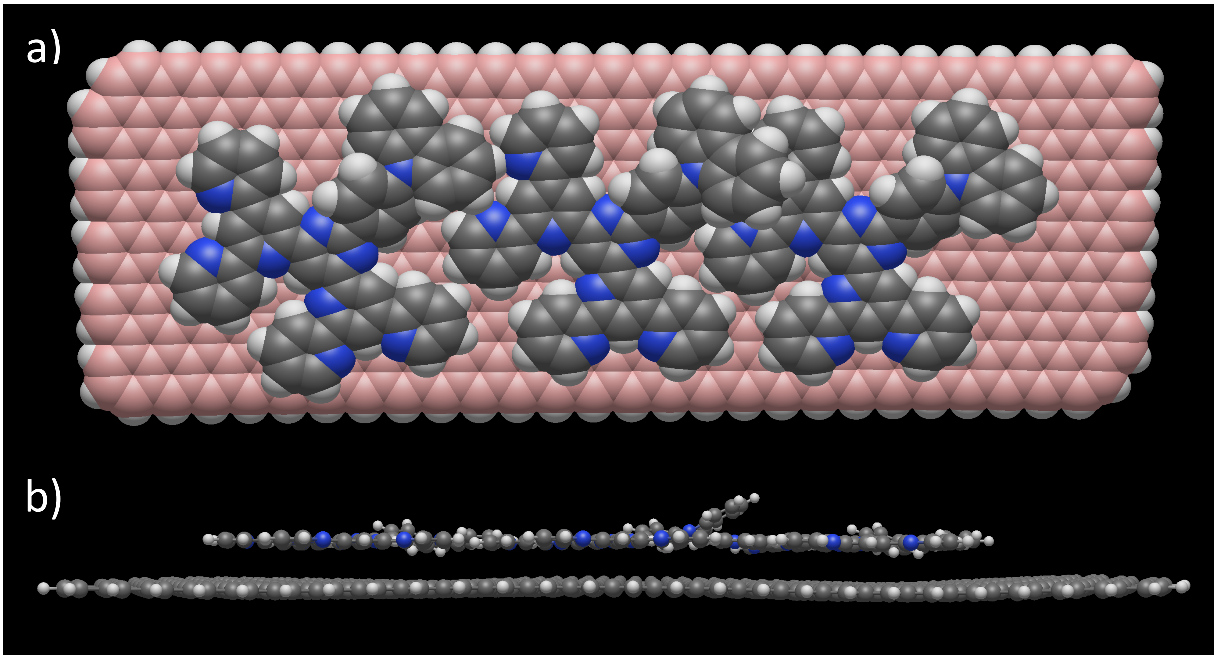

Figure S 11. Energy optimized structure of a trimer with one π overlap after optimization, a) top view (the carbon atoms of the graphene layer are colored for better visibility), b) side view with the protruding carbazole unit only of the molecule in the middle due to the π overlap.

7.2 Estimation of adsorption energy

The adsorption energy of a single BTP molecule on the graphene layer was determined to -399 kJ mol^{-1}.

The lateral displacement of a molecule with a protruding carbazole unit due to π overlap (configuration B) to a vdW-bound and fully planar adsorbed molecule (configuration A) amounts to around 0.4 nm. Based on the unit cell parameter of the dimer along the oligomer axis (4.16 nm, see Table 1 in main text), this means an additional increase of the unit cell by around 10%. Thus, the gain in adsorption energy of the reduced space due to the π overlap is -40 kJ mol^{-1}. Compared with the endothermic cost of the π overlap (+22 kJ mol^{-1}), the total gain of the π overlap is -18 kJ mol^{-1}.

8 References

[1] F. D. Goll, A. L. Schleper, A. J. C. Kuehne, U. Ziener, *J. Phys. Chem. C* **2020**, *124*, 20213-20221.

[2] J. Durinda, L. Szucs, L. Krasnec, J. Heger, V. Springer, J. Kolena, J. Keleti, *Acta Fac. Pharm. Bohem.* **1966**, *12*, 89-129.

[3] M. Miri, J. A. Morales-Tirado, *J. Chem. Educ.* **2003**, *80*, 839.

[4] OriginPro, Version 2019b, OriginLab Corporation, Northampton, MA, USA.

[5] C. Bannwarth, E. Caldeweyher, S. Ehlert, A. Hansen, P. Pracht, J. Seibert, S. Spicher, S. Grimme, *WIREs Comput. Mol. Sci.* **2020**, *11*, e1493.

[6] C. Bannwarth, S. Ehlert, S. Grimme, *J. Chem. Theory Comput.* **2019**, *15*, 1652-1671.

[7] S. Spicher, S. Grimme, *Angew. Chem. Int. Ed. Engl.* **2020**, *59*, 15665-15673.
